# ZnSe nanoparticles dispersed in reduced graphene oxides with enhanced electrochemical properties in lithium/sodium ion batteries†

**DOI:** 10.1039/c8ra03479f

**Published:** 2018-07-18

**Authors:** Xi Cao, Aijun Li, Yang Yang, Jitao Chen

**Affiliations:** College of Chemistry and Molecular Engineering, Peking University Beijing 100871 PR China xicao@pku.edu.cn; School of Earth and Space Science, Peking University Beijing 100871 PR China; Materials Science & Engineering, Columbia University New York NY USA

## Abstract

A ZnSe-reduced graphene oxide (ZnSe-rGO) nanocomposite with ZnSe dispersed in rGO is prepared *via* a one-step hydrothermal method and applied as the anode materials for both lithium and sodium ion batteries (LIBs/SIBs). The as-prepared composite exhibits greatly enhanced reversible capacity, excellent cycling stability and rate capability (530 mA h g^−1^ after 100 cycles at 500 mA g^−1^ in LIBs, 259.5 mA h g^−1^ after 50 cycles at the current density of 100 mA g^−1^ in SIBs) compared with bare ZnSe in both lithium and sodium storage. The rGO plays an influential role in enhancing the conductivity of the nanocomposites, buffering the volume change and preventing the aggregation of ZnSe particles during the cycling process, thus securing the high structure stability and reversibility of the electrode.

## Abstract 2

ZnSe-rGO nanocomposite with ZnSe dispersed in reduced graphene oxides is studied as an anode for lithium and sodium ion batteries (LIBs/SIBs).

## Graphical abstract

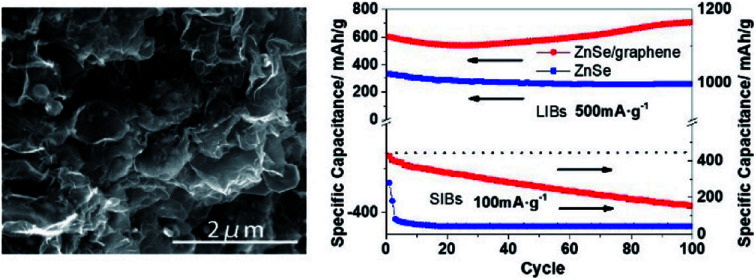

## 1. Introduction

To meet the increasing demand of various energy applications, such as grid storage, hybrid electric vehicles, and portable electronic devices, different kinds of rechargeable battery systems have been developed in recent years. Among them, lithium-ion batteries (LIBs) and sodium ion batteries (SIBs) are attracting great interest due to their higher energy density, longer cycling life, and environmental friendliness.^1–4^ Furthermore, due to the wide availability and low cost of sodium, ambient temperature sodium-ion batteries are considered to be the most promising electric energy storage system.^5–7^ However, considering the diameter of Na^+^ (0.106 nm), which is about 55% larger than that of Li^+^ (0.076 nm), it's still a great challenge and key issue to find suitable host materials for sodium ions.^8,9^ For example, graphite, the most commonly used anode in LIB systems, does not intercalate sodium to any appreciable extent.^10–12^

As the energy storage performance of LIBs and SIBs is strongly dependent on the electrochemical properties of electrode materials, a lot of efforts have been made to develop new electrode materials with high capacity and long cycling stability, such as transition metal oxides^13–15^ and Li/Na alloy-based materials,^16,17^ and among them, metal chalcogenides have attracted extensive investigation, and are well regarded as very potential electrode materials for both LIBs and SIBs owing to their attractive capacity. For instance, Kang *et al.* reported yolk-shell-structured MoSe_2_ microspheres prepared by the selenization of MoO_3_ microspheres, which delivered a stable capacity of 433 mA h g^−1^ for 50 cycles at a current density of 0.2 A g^−1^,^18^ different structures of nano MoS_2_ were also comprehensively studied as the anode for both lithium (with a capacity of about 1200 mA h g^−1^) and sodium ion batteries (with a capacity of about 400 mA h g^−1^).^19–22^ Besides, other metal chalcogenides including WS_2_ nanosheets,^23–25^ VSe_2_ nanoplates,^26^ SnS_2_ nanosheets,^27–30^ cobalt sulfide nanomaterials,^31,32^ and so on, were all studied as the anode materials for LIBs and SIBs due to their high specific capacitance. However, just like most of the metal oxides, metal chalcogenides also suffer from poor electrochemical properties, which have been mainly attributed to their low conductivity and the large volume change during charge and discharge processes. To address this problem, graphene has been widely used as a matrix to enhance the electrochemical performance due to its structural stability, superior electronic conductivity, material flexibility and large surface area. Significantly, except for being a buffer to accommodate the volume changes during reaction processes, graphene nanosheets also act as separators to effectively restrain the aggregation of particles upon long-term cycling.^30,31,33^

Recently, ZnS based nanomaterials were studied as the anode for both lithium and sodium ion batteries; for example, Wang *et al.* reported the ZnS/graphene nanocomposites as the anode materials for LIBs and exhibited a high capacity of 570 mA h g^−1^ after 200 cycles at a current density of 200 mA g^−1^.^33^ Another ZnS/porous carbon exhibited excellent electrochemical performance with a reversible capacity of 438 mA h g^−1^ in 300 cycles at 100 mA g^−1^.^34^ ZnS nanospheres showed a stable capacity of 480 mA h g^−1^ at a current density of 160 mA g^−1^ for SIBs.^35^ When ZnS nanoparticles were embedded in reduced graphene oxide, a maximum specific capacity of 481 mA h g^−1^ at the current of 100 mA g^−1^ after 50 cycles in SIBs was obtained. Similar to ZnS, zinc selenide (ZnSe) has the same crystal structure refined to the *F*4̄3*m* space group, fcc symmetry, with lattice parameters of *a* = *b* = *c* = 5.668 Å. Pyramidal [ZnSe_4_] units are arranged in individual vertex-sharing chains to form a tunnel structure along the [110] direction, which is an ideal space for accommodating Li and Na ions. Moreover, compared with ZnS, the ZnSe has more advantages including smaller band gap (2.7 eV, smaller than 3.54 eV of ZnS) and weaker bond strength due to the lower electronegativity of Se, indicating its better electric conductivity and easier reaction with Li^+^ and Na^+^, making the ZnSe-based material a promising anode for lithium and sodium storage. A ZnSe/C nanocomposite synthesized by solid-state routes showed the initial discharge capacity of 855 mA h g^−1^ in lithium ion batteries.^36^ ZnSe-reduced graphene oxide nanocomposites prepared by Zhang demonstrated a high reversible capacity of 876 mA h g^−1^ at 100 mA g^−1^ after 50 cycles.^37^ However, even such progresses have been made, as far as we know, the reports about ZnSe for the application of LIBs are still negligible compared with other metal chalcogenides. Meanwhile, to the best of our knowledge, the application of ZnSe as anode for sodium ion batteries has not been studied yet.

Herein, ZnSe-rGO nanocomposite with ZnSe nanoparticles dispersed in rGO is synthesized in a one-step hydrothermal method and studied as the anode materials for both LIBs and SIBs. Remarkably, compared with the bare ZnSe nanoparticles, ZnSe-rGO nanocomposite delivers enhanced reversible specific capacity, excellent cycling stability at the current density of 500 mA g^−1^ in LIBs (530 mA h g^−1^ after 100 cycles at 500 mA g^−1^). Meanwhile, by dispersing ZnSe nanoparticles in rGO, the cycling stability and rate performance are also greatly enhanced in SIBs (259.5 mA h g^−1^ after 50 cycles at the current density of 100 mA g^−1^).

## 2. Experimental

### 2.1 Synthesis

Graphite oxide (GO) is synthesized from natural graphite powders by using the modified Hummers method.^38^ For preparing ZnSe-rGO nanocomposites, 1.5 mmol Zn(NO_3_)_2_·6H_2_O and 1.5 mmol SeO_2_ are added into aqueous GO suspension (16 ml, 1.5 mg ml^−1^) which is pretreated with ultrasonication, after stirring for 10 minutes, 2 ml N_2_H_4_·H_2_O (80 wt% in water) is added into the mixture. After dispersing for 0.5 h, the mixed solution is then heated to 180 °C in a Teflon-lined autoclave (30 ml capacity) and kept at 180 °C for 24 h. The precipitates are cooled down to room temperature naturally and the black product is collected by centrifugation, then washed several times with ethanol and deionized water. After drying at 60 °C for 12 h, the final product is obtained. Bare ZnSe is prepared using the above method without adding GO. RGO networks is also obtained using above hydrothermal method without ZnSe.

### 2.2 Structural analyses

The crystal structure of the obtained sample is characterized by means of X-ray diffraction (XRD, D8 Bruker X-ray diffractometer with Cu-Kα radiation (*λ* = 1.5418 Å)) within the range of 6–60° (2*θ*). The morphology is examined using scanning electron microscopy (SEM, FEI, XL30 Sirion FEG). The amount of rGO in the composite is determined from the ignition loss of the sample at 800 °C in air with a thermo gravimetric (TGA) apparatus (Q50, TA).

Nitrogen adsorption–desorption isotherms are measured using Quantachrome NOVA 4200e system. Samples are degassed at 150 °C overnight under vacuum prior to measurements. The specific surface area and pore size distribution are determined by multipoint Brunauer–Emmett–Teller (BET), Barrett–Joyner–Halenda (BJH) desorption analyses, respectively.

### 2.3 Electrochemical measurements

To evaluate the electrochemical performance, a sandwich-type two-electrode testing cell is assembled using the as-prepared samples. The electrode slurry is prepared by mixing the as-synthesized ZnSe-rGO composites, Super P conductive carbon (TIMCAL Graphite & Carbon), and poly(vinylidene difluoride) (PVDF, Sigma-Aldrich) binder dispersed in an *N*-methyl-2-pyrrolidone (NMP, Alfa Aesar) solution at a weight ratio of 80 : 10 : 10, respectively. The as-prepared active material slurry is uniformly spread onto Cu foil and dried in a vacuum oven at 80 °C overnight prior to coin cell assembly. The mass loading of the electrode is about 0.8 mg cm^−2^. CR2016-type coin cells are assembled in an argon-filled glovebox (Innovative Technology, IL-2GB). For the lithium ion batteries, pure lithium foil is used as the counter electrode, and polypropylene film as the separator (Celgard 2400), 1 M LiPF_6_ solution in a volume ratio of 1 : 1 : 1 mixture of ethylene carbonate (EC)/dimethyl carbonate (DMC)/dimethyl ethylene carbonate (DEC) is used as the electrolyte, with 1% vinylene carbonate (VC) as the additive. For the sodium ion batteries, pure sodium foil is used as the counter electrode, and glass fiber (Whatman GF/A, grade) as the separator, 1 M NaClO_4_ solution in a volume ratio of 1 : 1 mixture of propylene carbonate/ethylene carbonate (EC) is used as the electrolyte, with 5% fluoro-ethylene-carbonate (FEC) as the additive.

Galvanostatic discharge and charge measurements are performed with LAND CT2001A tester (Wuhan, China) at different current densities in the voltage range of 0.01–3.5 V *versus* Li/Li^+^, and 0.01–3.0 V *versus* Na/Na^+^. Cyclic voltammetry (CV) is conducted on an electrochemical analyzer (CH Instruments, model 605C) in the voltage range of 0.01–3.0 V (*vs.* Li/Li^+^ or Na/Na^+^) at a scan rate of 0.2 mV s^−1^. Alternating current (AC) impedance is recorded by applying the Solartron 1287A in conjunction with a Solartron 1260FRA/impedance analyzer with amplitude of 5.0 mV in the frequency range from 100 kHz to 0.01 Hz.

## 3. Results and discussion

### 3.1 Structural properties

**Fig. 1:**
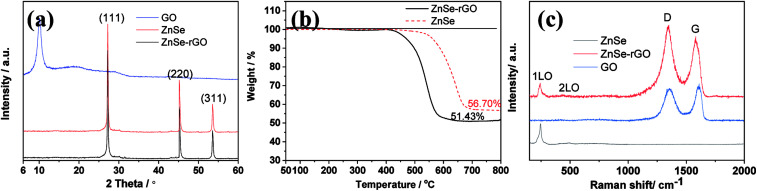
(a) XRD patterns of the graphene oxide, ZnSe, and ZnSe-rGO; (b) TGA curve of ZnSe and ZnSe-rGO from 25 °C to 800 °C at a heating rate of 10 °C min^−1^ in flow air; (c) Raman spectra of GO, ZnSe and ZnSe-rGO.

The diffraction patterns of graphene oxide, pure ZnSe, and ZnSe-rGO nanocomposite are shown in Fig. 1a, the peaks at 27.2, 45.2 and 53.66° are correspond to the (111), (220) and (311) crystal planes of ZnSe, respectively, which is in accordance with the ZnSe standard card (JCPDS 65-9602). TGA is carried out in flow air with a heat rate of 10 °C to determine the rGO content in the ZnSe-rGO composite, with the results shown in Fig. 1b. This figure compares the weight losses of pure ZnSe and ZnSe-rGO nanocomposites, the rGO content in ZnSe-rGO nanocomposites can be calculated to be 9.3% according to the TGA results (the calculation part can be found in ESI†). Fig. 1c gives the Raman spectra of bare ZnSe, GO and ZnSe/rGO nanocomposites. The D bond and G bond can be clearly detected in the GO and ZnSe-rGO composite, while the 1LO and 2LO modes of ZnSe can be found in both bare ZnSe and ZnSe-rGO, which confirms the existence of rGO in the nanocomposites.

**Fig. 2:**
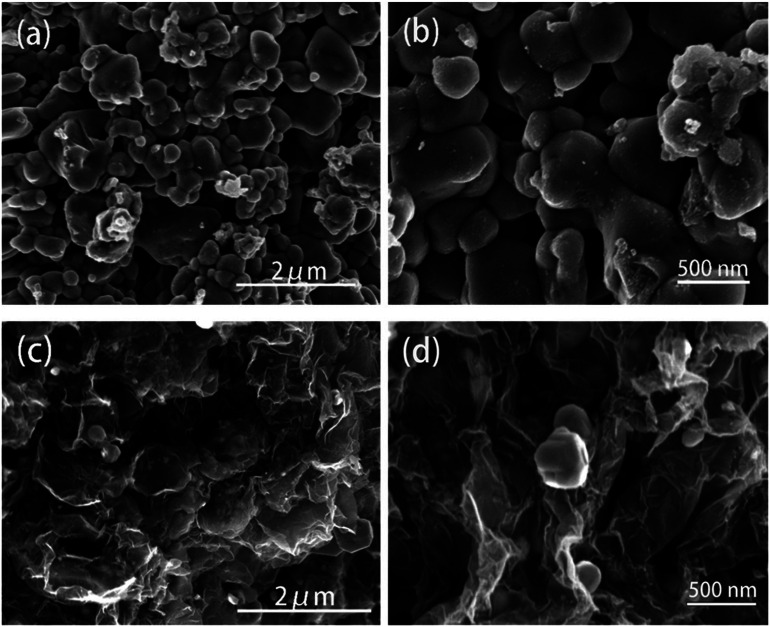
FESEM images of the ZnSe (a and b) and ZnSe-rGO (c and d).

The ZnSe without rGO exhibits a spherical-like morphology with the particle size ranging from 200 nm to 1000 nm and with some agglomeration, as shown in Fig. 2a and b, while the ZnSe particles in the ZnSe-rGO are appreciably smaller and with no aggregation (Fig. 2c and d), which suggests that the rGO hinder the ZnSe particle aggregation. In the same time, the ZnSe nanoparticles could also prevent the agglomeration of the rGO nanosheets.

**Fig. 3:**
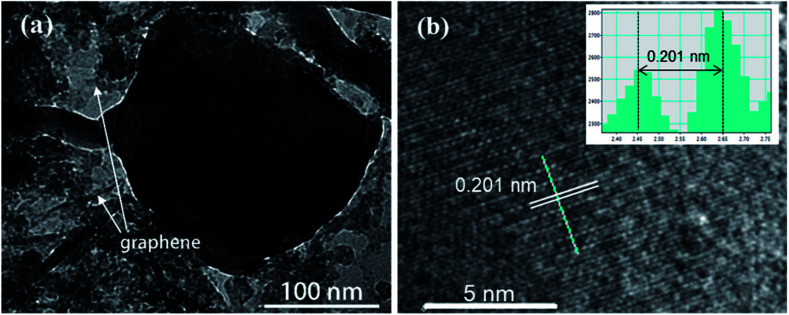
(a) TEM image and (b) high-resolution TEM image of the ZnSe-rGO nanocomposites. The lattice fringes can be indexed to ZnSe (JCPDS 65-9602).

Transmission electron microscopy (TEM) is also carried out to provide further insight into the morphology and structure of the resulting ZnSe-rGO composite. As shown in Fig. 3a, the dark particle with diameter of about 200 nm is ZnSe, with surrounded by the rGO nanosheets, which is in consistence with the SEM results. More detailed structural information can be found from the HRTEM image in Fig. 3b, where the lattice fringes of ZnSe nanoparticles can be clearly observed, suggesting the presence of a well-defined crystal structure. The periodic lattice fringe spaces are found to be around 0.201 nm corresponding to the interplanar spacing of (220) planes, in consistence with the XRD result.

**Fig. 4:**
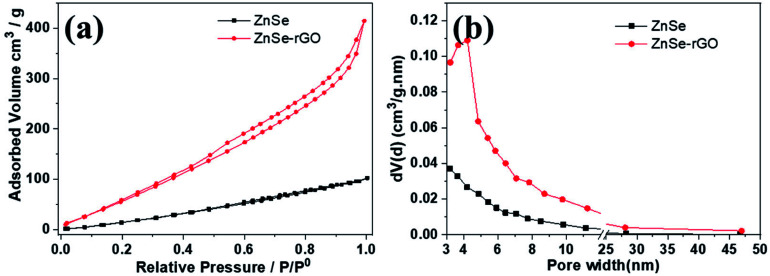
Nitrogen adsorption–desorption isotherms (a) and the corresponding BJH desorption pore size distribution (b) of ZnSe and ZnSe-rGO.

Nitrogen adsorption measurement is used to determine the pore structure of the ZnSe and ZnSe-rGO. Fig. 4 gives their adsorption–desorption and the pore size distribution curves. Both samples exhibit type IV isotherms with a typical H4 type hysteresis loop, which can be attributed to the mesoporous structures formed by slit-like pores and the aggregation of particles.^39^ The ZnSe nanoparticles exhibit a broad pore size distribution from 3–30 nm, indicating the existence of mesopores originated from the aggregation of particles. The Brunauer–Emmett–Teller (BET) derived surface area is determined to be 52.39 m^2^ g^−1^, with a total pore volume of 0.142 cm^3^ g^−1^. In comparison, ZnSe-rGO nanocomposite exhibits a slight increase of the surface area to 85.6 m^2^ g^−1^, with the pore volume increases to 0.637 cm^3^ g^−1^ at the same time. The ZnSe nanocomposite demonstrates a maximum pore size distribution peak at 3–5 nm (Fig. 4b), which might be due to the space created by the intercalated rGO nanosheets.

### 3.2 Electrochemical performance for LIBs

**Fig. 5:**
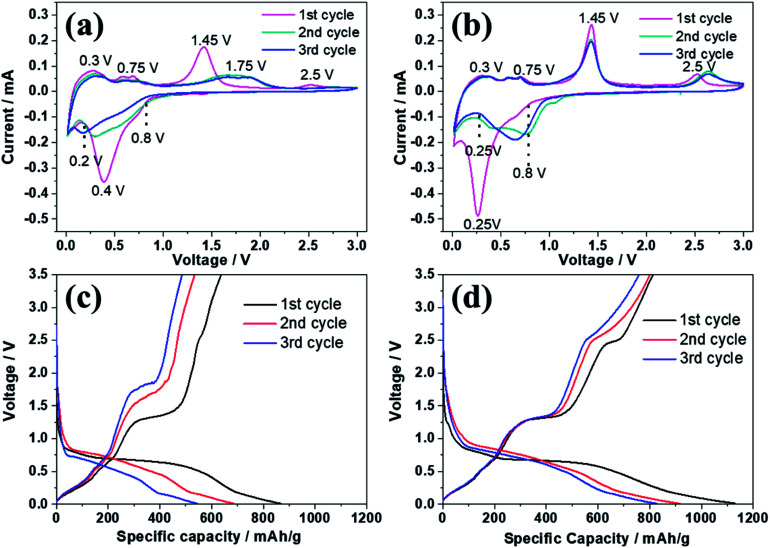
CV curves of ZnSe (a) and ZnSe-rGO (b) electrode measured in the voltage range of 0.01–3.0 V (*vs.* Li/Li^+^); first three discharge and charge curves of ZnSe (c) and ZnSe-rGO (d) electrode at the current density of 100 mA g^−1^.

Fig. 5 reveals the electrochemical properties of bare ZnSe and ZnSe-rGO as anodes in lithium ion batteries. The cyclic voltammograms (CVs) of the samples for the first three cycles are displayed in Fig. 5a and b. In the first discharge curve of bare ZnSe, the long slope from about 0.8 V to 0.27 V is related with the formation of the SEI layer and the conversion of ZnSe into Zn and Li_2_Se.^36,37^ An obvious reduction peak observed at below 0.2 V is associate with the formation of Li–Zn alloy such as LiZn_4_, Li_2_Zn_5_, LiZn_2_, Li_2_Zn_3_. According to the literature, when the electrode is fully discharged close to 0.0 V, the Li_*x*_Zn alloy further converts to the pure LiZn.^36,37,40^ The reduction peak of ZnSe-rGO are not repeatable and shift to one flat peak at around 0.6 V after the first cycle, which might be due to some irreversible reactions with structure changes and the partially irreversible formation of Li_2_Se and LiZn alloy only in the first discharge process.^40^ During the charge process, the small oxidation peaks from 0.3–0.75 V are related with the dealloying process of Li–Zn alloy,^40^ the next obvious oxidation peak at about 1.45 V is associated with the regeneration of ZnSe from Zn and Li_2_Se. The belonging of the oxidation peak at around 2.5 V still remains unclear in literature even the similar phenomenon appears in the ZnS based LIBs,^33,36,37^ it might be due to the conversation of Li_2_Se into Se:^41^

1 ZnSe → Li_2_Se + Zn → Li_2_Se + Li_*x*_Zn → Li_2_Se + LiZn

2 Li_2_Se + LiZn → Li_2_Se + Li_*x*_Zn → Li_2_Se + Zn → ZnSe

3 Li_2_Se ↔ 2Li^+^ + Se +2e^−^

CV curves of ZnSe-rGO are better overlapped in the subsequent scans, indicating the material has a higher reversibility and better stability for the lithiation and delithiation. However, as for the ZnSe electrode, the obvious oxidation peak at about 1.4 V associated with the regeneration of ZnSe from Zn and Li_2_Se becomes weak and move to a higher voltage from the 2^nd^ cycle, indicating a poor reversibility and an increased electrochemical polarization. The small oxidation peaks related with the dealloying process of Li–Zn and the long obvious reduction becomes weak from the 2^nd^ cycle, indicates the poor cycling performance.

The electrochemical performance of the samples are further evaluated by charge–discharge curves obtained at 100 mA g^−1^ as shown in Fig. 5c and d. For both samples, there are two obvious plateaus located at around 0.8–0.3 and 0.27 V on the first discharge curves, and an obvious plateau located at around 1.2 V on the first charge curves, which is in accordance with the CV profiles. The initial discharge capacity of the ZnSe-rGO is 1132 mA h g^−1^, while the initial charge capacity reaches 813 mA h g^−1^, with an initial coulombic efficiency of 71.8%. The capacity loss should be associated with the formation of SEI layer on the electrode surface and the volume change of the active materials, which is common for the anode materials. The coulombic efficiency rises to 92% in the 3rd cycle, and remains at approximately 99% in the following cycles. The high coulombic efficiency indicates a stable SEI film on the electrode surface. However, even the first charge capacity of ZnSe only reaches 637 mA h g^−1^, and the corresponding coulombic efficiency is calculated to be 73.48%, the rapid capacity decay occurs in the following cycles.

**Fig. 6:**
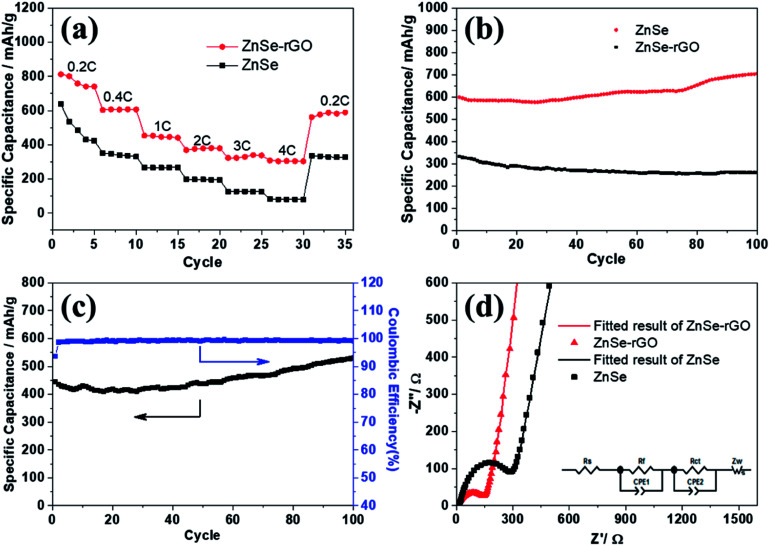
(a) Rate capability of ZnSe and ZnSe-rGO at different current densities (1C = 500 mA g^−1^); (b) cycling performance of ZnSe and ZnSe-rGO at 0.4C; (c) cycling performance of ZnSe-rGO at 1C; (d) EIS plots of ZnSe and ZnSe-rGO at 25 °C (inset: equivalent electrical circuit model).

The rate performance of the two electrodes is evaluated and compared. As shown in Fig. 6a, ZnSe-rGO is found to demonstrate higher capacities than ZnSe at different current densities. For example, a reversible capacity of approximately 740 mA h g^−1^ is achieved at the current density of 100 mA g^−1^, when current density increases to 200 and 500 mA g^−1^, the charge capacities are obtained to be 606 and 446 mA h g^−1^. Afterwards, the charge capacity returns to 590 mA h g^−1^ when the current density switches back to 100 mA g^−1^. However, bare ZnSe delivers much lower capacities, for example, ZnSe delivers only 423 mA h g^−1^ even at the low current density of 100 mA g^−1^. Fig. 6b shows the cycling performance of ZnSe and ZnSe-rGO electrodes at 200 mA g^−1^ for 100 cycles. As we can see, the ZnSe-rGO demonstrates a higher reversible capacity and excellent cycling stability, compared with the bare ZnSe electrode, with an initial charge capacity of *ca.* 600 mA h g^−1^ and increased to 705 mA h g^−1^ after 100 cycles, while the bare ZnSe electrode delivers an initial specific capacity of only 332 mA h g^−1^ and declined to about 260 mA h g^−1^ after 100 cycles. Considering the 9.3% of rGO in the ZnSe-rGO nanocomposites (with the specific capacitance of rGO to be 440 mA h g^−1^, as shown in Fig. S1a†), it can be calculated that the corresponding capacity of ZnSe should be 732.2 mA h g^−1^.

High rate cycling performance of ZnSe-rGO at 500 mA g^−1^ is measured and displayed in Fig. 6c, the capacity first decreases from 445 to 410 mA h g^−1^ in the first 15 cycles and rises stably to 530 mA h g^−1^. Meanwhile, the coulombic efficiency of the ZnSe-rGO reaches to nearly 100% after first several cycles, further demonstrating the excellent high rate cycling performances of the ZnSe-rGO nanocomposites. Fig. 6d shows the Nyquist plots of the ZnSe-rGO and ZnSe electrodes after 100 cycles (at 200 mA g^−1^). Both profiles displays a depressed semicircle in the high frequency region associated with the combined process of surface film (*R*_f_) and the charge transfer resistance (*R*_ct_), and a long slope line represents the Warburg impedance (*Z*_W_) at low frequency, which indicates the diffusion of lithium ions in the solid matrix.^45^ The plots are fitted with the equivalent electrical circuit model (as shown in inset Fig. 6d), in which the symbols, *R*_s_, *R*_f_, *R*_ct_ and *Z*_W_, represents the solution resistance, contact resistance, charge-transfer resistance and Warburg impedance, respectively. The fitting results are shown in Table 1. The *R*_f_ and *R*_ct_ values of ZnSe-rGO electrode are 15.8 Ω and 157.2 Ω, respectively, which are much smaller than those of the ZnSe electrode (176.2 Ω and 194 Ω), suggesting that the rGO significantly lowers contact and charge-transfer resistance.

**Table 1:** Impedance parameters calculated from equivalent circuit (LIBs)

Sample	*R*_s_ [Ω]	*R*_f_ [Ω]	*R*_ct_ [Ω]
ZnSe	5.3	176.2	194
ZnSe-rGO	4.9	15.8	157.2

**Fig. 7:**
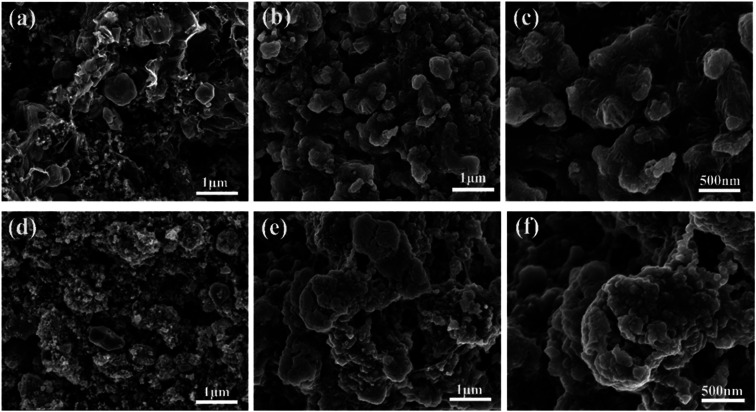
SEM images of the electrodes before cycling: ZnSe-rGO (a), ZnSe (d) and after 100 cycles in LIBs: ZnSe-rGO (b) and (c), ZnSe (e) and (f).

The rGO matrix significantly improves both rate performance and cycling stability by improving the conductivity of nanocomposites, buffering the volume changes of ZnSe nanoparticles during cycles, and acting as a spacer to effectively restrain the aggregation of ZnSe nanoparticles. The rGO network with a large specific surface may facilitate the diffusion and transportation of electrolytes within the electrodes for fast redox reactions during the charge–discharge process at high current density, and at the same time dilute the local current density. As for the capacity-climbing phenomenon, which is common in the field of Li-based rechargeable batteries, and can be found in other anodes.^42,43^ There are several possible reasons concerning this phenomenon, first, as described in previous work, this “extra capacity” is due to the reversible growth of a polymeric gel-like which is built up continually over a number of cycles.^44^ Besides, the slow increase in capacity may be attributed to the electrochemical activation of the ZnSe nanoparticles which generates more defects and active sites for reaction. More work still need to be done to give a clear explanation for this phenomenon. However, for the bare ZnSe, after the first lithiation/delithiation process, the particles may experience a large volume change and pulverize into small parts, thus the contact between active particles may also decrease, and the SEI layer may also continue forming on the surface of the small parts, along with the increase of resistance. Above predictions can also be evidenced by the SEM results of the structure change of both ZnSe-rGO and ZnSe electrode after 100 charge/discharge cycles. As shown in Fig. 7b and c, even after 100 cycles, the ZnSe in rGO still retain the original morphology (Fig. 7a) without any fracture. Also, there is a thin gel-like layer on the surface of the electrode after 100 cycles for the ZnSe-rGO electrode, which is not observed on the bare ZnSe electrodes, which may be related to the increased capacity. In contrast, as shown in Fig. 7e and f, the ZnSe particles are completely destroyed after 100 charge/discharge, compared with that of before cycling (Fig. 7d).

### 3.3 Electrochemical performance for SIBs

**Fig. 8:**
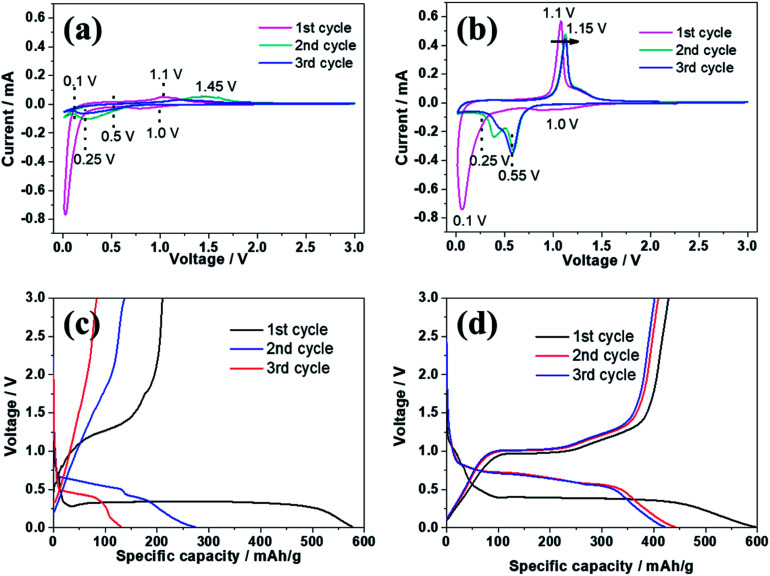
CV curves of ZnSe (a) and ZnSe-rGO (b) electrode measured in the voltage range of 0.01–3.0 V (*vs.* Na/Na^+^); first three discharge and charge curves of ZnSe (c) and ZnSe-rGO (d) electrode at the current density of 50 mA g^−1^.

Fig. 8 reveals the electrochemical properties of bare ZnSe and ZnSe-rGO electrodes as anodes in sodium ion batteries. The CV curves of both samples are recorded in a voltage range of 0.01–3.0 V at a scan rate of 0.2 mV s^−1^. As we can see from Fig. 8a and b, in the first cathodic scanning process, one broad at around 1.0 V occurs for both electrodes, which is associated with the insertion of sodium-ion and the formation of solid electrolyte interphase (SEI) layer due to the decomposition of electrolyte.^46^ Another strong cathodic peak appears below 0.25 V is related with the conversion of ZnSe into Zn and Na_2_Se, followed with the formation of Na–Zn alloy.^46,47^ This peak changes to be a broader peak and shifted to a more positive potential region in the second negative scan, indicating that the electrochemical polarization increases in the second sodiation process. From the second discharge process, the small peak under 0.1 V is related to the formation of Na–Zn alloy, and the broad peak from 0.5–0.1 V is referred to the conversion of ZnSe into Zn and Na_2_Se. The peak intensity of the conversion reaction is greater than that of the alloy reaction, implying that the conversion reaction contributes the majority of the reversible capacity. During the charge step, the obvious oxidation peak at about 1.1 V is associated with the regeneration of ZnSe from Zn and Na_2_Se.^46^ According to the analysis above, the basic reactions of ZnSe with Na can be expressed by the following equations:

4 ZnSe + 2Na^+^ + 2e^−^ ↔ Na_2_Se + Zn

5 13Zn + Na^+^ + e^−^ ↔ NaZn_13_

The alloying reaction (5) between the zinc and sodium can be obtained according to the Na–Zn phase diagram and has been experimentally confirmed.^47^ There is a clear difference of the CV curves between ZnSe and ZnSe-rGO electrode, as shown in Fig. 8a and b, both of the cathodic and anodic peak intensity of the ZnSe-rGO are stronger than those of ZnSe, which indicates a lower resistance and an easier Na^+^ diffusion in the ZnSe-rGO. The oxidation peak of ZnSe-rGO slightly moves to 1.15 V from the second cycle while that of ZnSe electrode moves obviously to 1.45 V, which indicates the higher electrochemical polarization of the bare ZnSe electrode. Besides, the CV curves of ZnSe-rGO are better overlapped after the first scan, indicating the material has a better reversibility and stability for the sodiation/desodiation.

Fig. 8c and d exhibits the galvanostatic charge–discharge curves at 50 mA g^−1^ in a voltage range of 0.01–3.0 V, for both samples, there is an obvious plateau located at around 0.27 V on the first discharge curves, and an obvious plateau located at around 1.1 V on the first charge curves, which is in accordance with the CV profiles. In the following cycles, there are two obvious typical charge–discharge plateaus, corresponding to the insertion/extraction of sodium ion into/from the electrodes. The initial discharge capacity of the ZnSe-rGO is 596.5 mA h g^−1^, while the initial charge capacity is 428.3 mA h g^−1^, with an initial coulombic efficiency of 71.8%. The coulombic efficiency rises to 92% in the 3^rd^ cycle, and remains above 95% in the following cycles. However, the first charge capacity of ZnSe only reaches 280 mA h g^−1^, with the corresponding coulombic efficiency is calculated to be 48.5%. Moreover, the ZnSe exhibits severe capacity fading after the first cycle, which is in consistence with the lithium ion batteries.

**Fig. 9:**
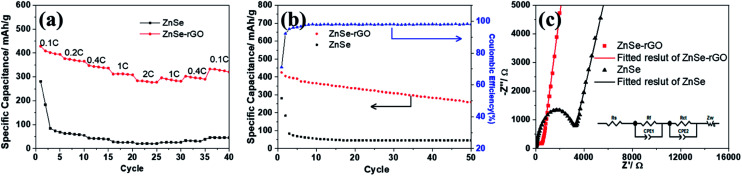
(a) Rate capability of ZnSe and ZnSe-rGO at different current densities (1C = 500 mA g^−1^); (b) cycling performance of ZnSe and ZnSe-rGO at 0.2C; (c) EIS plots of ZnSe and ZnSe-rGO at 25 °C (inset: equivalent electrical circuit model).

As shown in Fig. 9a, the charge capacities of the ZnSe-rGO composite electrode are 393.9, 365.6, 337 and 310 mA h g^−1^ at corresponding discharge–charge current densities of 50, 100, 200, 500 mA g^−1^, respectively. Even at a high current density of 1000 mA g^−1^, it still retains a high capacity of 277 mA h g^−1^. When the current density is reversed back to 50 mA g^−1^ after different rate testing, the charge capacity returns back to 320 mA h g^−1^, indicating the high reversibility of the ZnSe-rGO electrode. These capacities are all higher than ZnSe electrode. As we can see, ZnSe delivers only 70 mA h g^−1^ after several cycles even at the low current density of 50 mA g^−1^.

The cycling performances were compared at a current density of 100 mA h g^−1^, as shown in Fig. 9b. As displayed, the pure ZnSe demonstrates a poor cycling stability with the quick decrease of specific capacity to 45 mA h g^−1^ after just 15 cycles from the initial reversible value of 280.5 mA h g^−1^, with a poor capacity retention of only 16.0%. However, after dispersing these ZnSe nanoparticles in the rGO, the cycling stability is greatly enhanced. After 50 galvanostatic charge–discharge cycles, the specific capacity remains 259.5 mA h g^−1^ for ZnSe-rGO electrodes, with capacity retention of 69.3%. The corresponding capacity contributed by ZnSe should be 270 mA h g^−1^ by subtracting the capacity contributed by rGO (Fig. S1b†). We believe that the cycling stability can be further enhanced by adjusting the ratio of rGO added into the composites. Pan *et al.* reported their work on ZnS/rGO nanocomposites with different rGO content as the anode materials in sodium-ion batteries, the results showed that the nanocomposites with a rGO content of 31% exhibited the best sodium storage properties, including the highest capacity and best cycling stability.^46^ We designed the different contents of rGO to optimize the experimental results. The materials are synthesized using the same procedure except the amount of GO added to be 54 mg and 90 mg, the obtained materials are denoted as ZnSe-rGO-2 and ZnSe-rGO-3. The rGO content in above two materials can be obtained from the TGA results, as shown in Fig. S2a,† which are calculated to be about 20.3% and 32.9%. The electrochemical performance of these two materials were tested and compared. As shown in Fig. S2b and c,† both materials exhibit enhanced rate and cycle performance, and ZnSe-rGO-3 behaves lower capacitance but better cycling performance, which might due to the higher graphene content. After 50 cycles, the specific capacity remains 321.2 and 310.1 mA h g^−1^ for ZnSe-rGO-2 and ZnSe-rGO-3 electrode, with capacity retention of 83.0% and 94.5%, respectively.

Fig. 9c shows the Nyquist plots of the ZnSe-rGO and ZnSe electrodes after 50 cycles (at 100 mA g^−1^), which is similar to the lithium ion batteries. The plots are also fitted with the same equivalent electrical circuit model (as shown in inset Fig. 9c). The fitting results are shown in Table 2. The *R*_f_ and *R*_ct_ values of ZnSe-rGO electrode are 292.3 Ω and 365.9 Ω, respectively, which are much larger compared with the those in lithium ion batteries, indicating the higher resistance of sodium ion batteries. But they are still smaller than those of the ZnSe electrode (2019 Ω and 966.4 Ω) in SIBs, suggesting that the contact and charge-transfer resistance are greatly decreased after introducing the conductive rGO into the ZnSe particles, which also indicates a faster electrochemical reaction kinetics at the electrode/electrolyte interface that will finally contribute to the enhancement of sodium-ion storage capacity of ZnSe-rGO composite.

**Table 2:** Impedance parameters calculated from equivalent circuit (SIBs)

Sample	*R*_s_ [Ω]	*R*_f_ [Ω]	*R*_ct_ [Ω]
ZnSe	18.3	2019	966.4
ZnSe-rGO	20.1	292.3	365.9

**Fig. 10:**
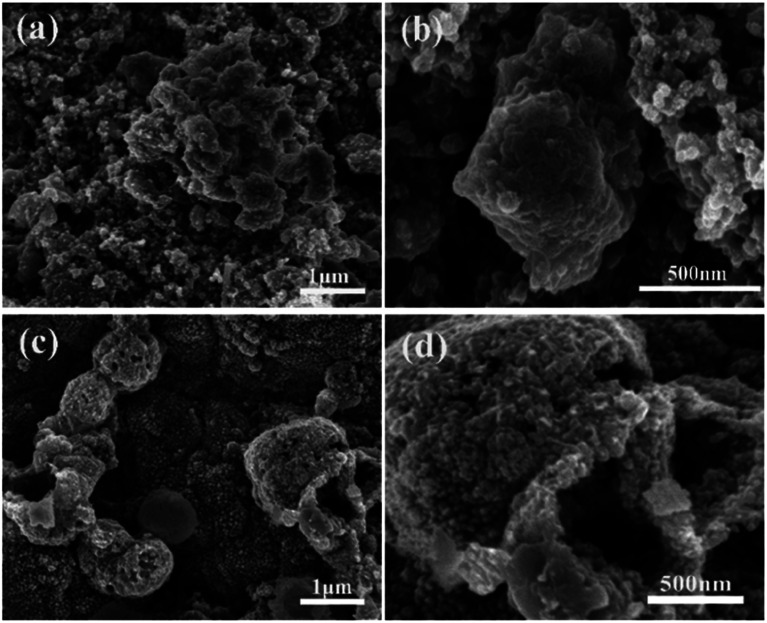
SEM images of the electrodes after 100 cycles in SIBs: ZnSe-rGO (a) and (b), ZnSe (c) and (d).

**Fig. 11:**
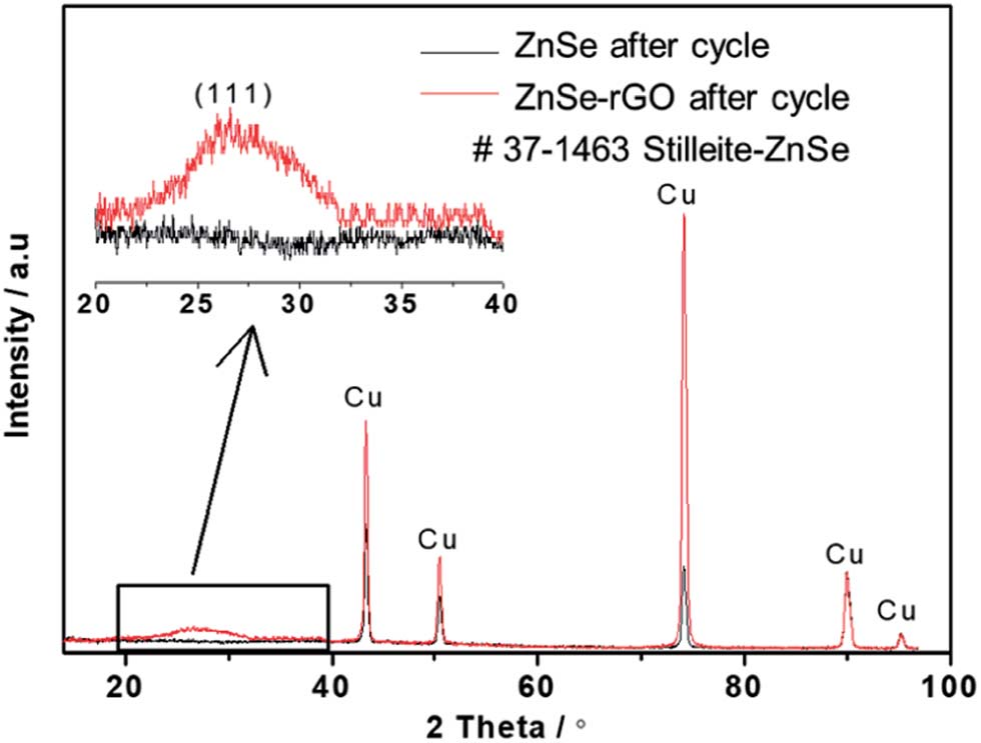
XRD pattern of the two electrodes and after 100 cycles in SIBs.

SEM investigation is also employed to study the structure changes of both ZnSe-rGO and ZnSe electrodes after 50 charge/discharge cycles in sodium ion batteries. As shown in Fig. 10a, ZnSe-rGO nanocomposites don't crack into small particles after cycling even with obvious volume expansion, which indicates a good stability of ZnSe-rGO during charge/discharge process. However, the bare ZnSe particles are completely destroyed after 50 charge/discharge, as we can see from Fig. 10b, the particles are completely cracked into parts and the smooth surface of the particles are destroyed, compared with that of before cycling (Fig. 2b). The *ex situ* XRD is also employed to study the two electrodes after fully charged to 3.0 V, as shown in Fig. 11, the diffraction peaks of ZnSe in the bare ZnSe electrode cannot be observed after cycling, while the ZnSe-rGO composites still exhibits a strong the strong (111) diffraction peak. This results confirms the fact that the rGO in the ZnSe-rGO composites helps to keep the ZnSe stable and reversible during cycles.

## 4. Conclusions

In summary, the ZnSe-rGO nanocomposite is synthesized *via* a one-step hydrothermal method and studied as an anode material for both LIBs and SIBs. The rGO in the nanocomposites greatly enhances the conductivity of nanocomposites, buffers the volume change and prevented the aggregation of ZnSe particles during the cycling process, and thus guarantees the structure stability and reversibility of the electrode. The synergetic effects between ZnSe nanoparticles and rGO make ZnSe-rGO nanocomposites exhibit enhanced reversible capacity, excellent cyclic performance and rate capability (530 mA h g^−1^ after 100 cycles at 500 mA g^−1^) compared with the bare ZnSe in LIBs. When tested as anode material in sodium ion batteries, the ZnSe-rGO shows a reversible capacity of 259.5 mA h g^−1^ after 50 cycles at the current density of 100 mA g^−1^, while the bare ZnSe nanoparticles only have a low reversible capacity of 45 mA h g^−1^.

## Conflicts of interest

There are no conflicts to declare.

## Supplementary Material

RA-008-C8RA03479F-s001
