# Supplementary material for: ZnSe nanoparticles dispersed in reduced graphene oxides with enhanced electrochemical properties in lithium/sodium ion batteries
Source: RSC Adv. 2018 Jul 18;8(45):25734–44. doi: 10.1039/c8ra03479f (PMC9082557; doi:10.1039/c8ra03479f)
Supplement: RA-008-C8RA03479F-s001 [file RA-008-C8RA03479F-s001.pdf]

Electronic Supplementary Material (ESI) for RSC Advances.
This journal is © The Royal Society of Chemistry 2018

Supporting Information:

ZnSe nanoparticles dispersed in reduced graphene oxides with enhanced electrochemical properties in lithium / sodium ion batteries

Xi Cao[a*], Aijun Li[b, c], Yang Yang[b], Jitao Chen[a]

a. College of Chemistry and Molecular Engineering, Peking University, Beijing 100871, PR China.

b. School of Earth and Space Science, Peking University, Beijing 100871, PR China.

c. Materials Science & Engineering, Columbia University, New York, NY, USA.

* Corresponding Author. E-mail: xicao@pku.edu.cn

Abstract: ZnSe- reduced graphene oxides (ZnSe-rGO) nanocomposite with ZnSe dispersed in rGO is prepared via a one-step hydrothermal method and applied as the anode materials for both lithium and sodium ion batteries (LIBs/SIBs). The as-prepared composite exhibits greatly enhanced reversible capacity, excellent cycling stability and rate capability (530 mAh/g after 100 cycles at 500 mA/g in LIBs, 259.5 mAh/g after 50 cycles at the current density of 100 mA/g in SIBs) compared with bare ZnSe in both lithium and sodium storage. The rGO plays an influential role in enhancing the conductivity of the nanocomposites, buffering the volume change and preventing the aggregation of ZnSe particles during cycling process, thus securing the high structure stability and reversibility of the electrode.

Keywords: Hydrothermal method; Lithium ion batteries; Sodium ion batteries; Anode material; ZnSe; rGO

The graphene content in ZnSe/rGO nanocomposites can be calculated according to the following equation:

$$\text{Graphene content} = 100\% * \left(1 - \frac{1 - Weight\ loss\ of\ ZnSe/graphene\ nanocomposite}{1 - Weight\ loss\ of\ ZnSe}\right) = 9.3\%$$

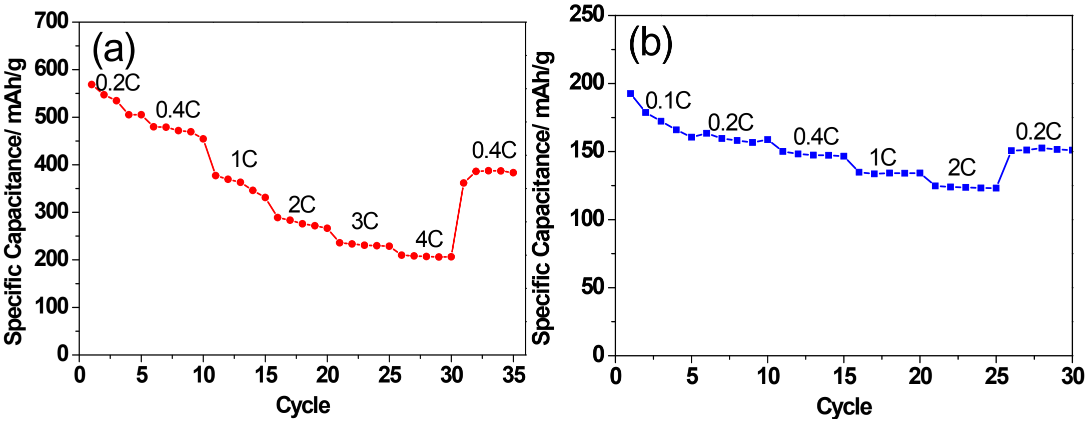

Fig. S1 Rate capability of rGO in lithium (a) and sodium (b) ion batteries

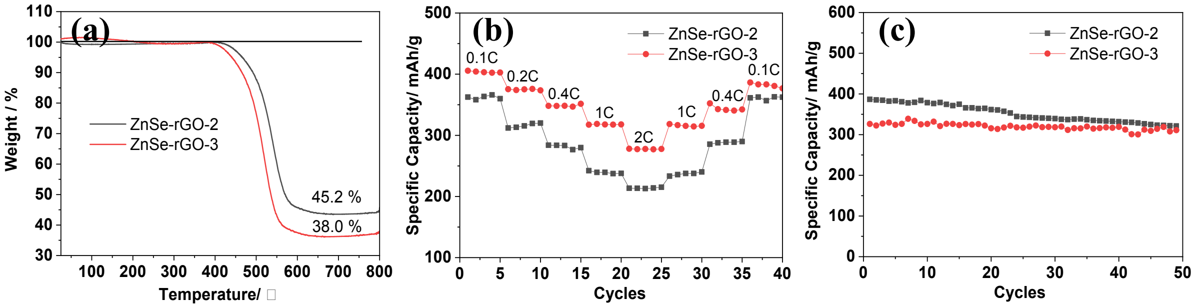

Fig. S2 TGA results of ZnSe-rGO-2 and ZnSe-rGO-3(a); Rate capability(b) and cycle performance(c) of ZnSe-rGO-2 and ZnSe-rGO-3 in sodium ion batteries
